# Junior doctors in their first year: mental health, quality of life, burnout and heart rate variability

**DOI:** 10.1007/s40037-013-0075-y

**Published:** 2013-08-09

**Authors:** Marcus A. Henning, John Sollers, Joanna M. Strom, Andrew G. Hill, Mataroria P. Lyndon, David Cumin, Susan J. Hawken

**Affiliations:** 1 Centre for Medical and Health Sciences Education, University of Auckland, Auckland, New Zealand; 2 Department of Psychological Medicine, Faculty of Medical and Health Sciences, University of Auckland, Auckland, New Zealand; 3 Counties Manukau District Health Board, Auckland, New Zealand; 4 Department of Surgery, South Auckland Clinical School, The University of Auckland, Auckland, New Zealand; 5 Department of Surgery, Faculty of Medical and Health Sciences, University of Auckland, Auckland, New Zealand

**Keywords:** Mental health, Heart rate variability, Doctors

## Abstract

There is a burgeoning interest in, and evidence of, quality of life and burnout issues among doctors. It was hypothesized that the junior doctors in this study would experience psychosocial and physiological changes over time, and that the obtained measures would indicate psychosocial and physiological anomalies. In addition, it was hypothesized that their psychosocial perceptions would be significantly associated with their physiological measures. A total sample of 17 junior doctors in their first year of training volunteered for this study. Over four time periods separated by 6 week phases, the doctors completed a set of quality of life and psychosocial inventories and wore a Polar RS800 Heart Rate Monitor over a day and night time interval. The findings showed that this sample of doctors did not report any problems associated with depression, anxiety, stress, burnout or quality of life (psychosocial measures). In addition, their heart rate variability scores (physiological measures) did not show any significant fluctuations. Furthermore, the responses from the self-report instruments measuring stress, anxiety, depression, quality of life and burnout did not consistently correlate with the HRV information suggesting a mind–body disconnection. More work needs to be done on larger samples to investigate these findings further given that the literature shows that junior doctors are likely to be stressed and working in stress-provoking environments.

## Introduction

Learning and working in the hospital environment is challenging, rewarding and exhausting. There is a burgeoning interest in, and evidence of, quality of life and burnout issues among doctors [1–3]. In a recent study [4], doctors’ stress responses were analysed in line with simulated bad-news consultations. Providing bad-news was considered a stress-provoking event; hence the authors linked psychosocial perceptions of depression, anxiety, stress, burnout and fatigue with physiological measures. The findings of the Brown et al. [4] study suggested that inexperienced and fatigued doctors were more likely to exhibit high levels of autonomic arousal compared to their more experienced peers.

The main thrust behind the present study was to investigate the well-being of junior doctors in their first year of studying and working in a public teaching hospital in New Zealand. To gain registration by the Medical Council of New Zealand, junior doctors at this hospital are required to satisfactorily complete at least four three month runs (rotations), two of which must be surgical and medical (category A). The remaining two runs can come from the category A or B list that consists of paediatrics, orthopaedics, general practice, psychiatry, oncology, emergency medicine, obstetrics and gynaecology, anaesthesia, neurosurgery, and/or general relieving duties [5]. The Medical Council of New Zealand [5] provide guidelines that structure the supervision and learning process so that these junior doctors can gain valuable clinical skills that can build on their prior training and learning. Supervision is provided by the doctor’s supervising consultant and registrar in the areas of clinical work.

Similar to the Brown et al. study [4], the junior doctors in this study were inexperienced and thus likely experiencing high levels of stress due to issues of workload, transitioning from the university to workplace, clinical performance, and receiving supervisor feedback. The heavy workloads being placed on junior doctors in their clinical environment creates a challenging and inspiring workplace but may also produce adverse stress, fatigue, and the potential for burnout [6, 7]. The Medical Council of New Zealand [5] have a strong mandate for supporting the personal health and wellbeing of interns working in a New Zealand hospital and this mandate created the rationale to measure the well-being of junior doctors being supervised in this local teaching hospital.

The findings from other researchers indicate that junior doctors may under-report their levels of stress [1, 2, 4]. One possible measure of physiologic stress that is objective and can be captured unobtrusively is heart rate variability (HRV) [8, 9]. To gain a more integrated perspective of the junior doctors’ experience we decided to obtain both psychosocial ratings (depression, anxiety, stress, burnout and quality of life perceptions) and physiological measures (HRV) with the aim of allaying problems associated with gathering purely self-report data, such as dishonesty, response bias, and memory inconsistencies [10].

It was hypothesized that the junior doctors in this study would experience psychosocial and physiological changes over time, and that the obtained measures would indicate psychosocial and physiological anomalies. In addition, it was hypothesized that their psychosocial perceptions would be significantly associated with their physiological measures.

## Method

Ethics approval from The University of Auckland Human Participant Ethics Committee was acquired (2010/526). All 33 junior doctors working at Middlemore Hospital in Auckland, New Zealand, were approached to be involved in the study.

Over four time periods separated by 6 week phases, the doctors completed a set of quality of life and well-being inventories and wore a Polar RS800 Heart Rate Monitor. On each occasion, HRV measures were obtained over a 24 h interval (from 9am to 11am and 2am to 4am). The POLAR system consists of a simple two lead ECG measured via a surface band placed around the participants’ thorax. The data were transmitted and stored on the wristwatch itself with further analysis and processing being accomplished offline. The data were analysed using KUBIOS Pro HRV analysis package [11]. The root mean square of the successive differences (rMSSD) was calculated as a measure of HRV according to the recommendations of the Task Force of the European Society of Cardiology and the North American Society of Pacing and Electrophysiology [12]. The rMSSD values were log-transformed for analysis as the data were skewed.

Three well cited measures of psychosocial well-being were used: The Depression, Anxiety and Stress Scales (DASS-21) [13], the Eurohis quality of life scale (EUROHIS-QOL 8-item) [14], and the Copenhagen Burnout Inventory (CBI) [15]. The DASS-21 has three scales (depression, anxiety and stress), the EUROHIS-QOL has one scale and the CBI has 3 scales (person, work and client), thus providing in total seven psychosocial measures.

The responses from the junior doctors were analysed using PASW (version 18). Due to the low *n*-values and non- Gaussian nature of the dataset, the Wilcoxon Signed-Rank Test was employed (Table 1) [16]. Pearson correlations were also computed between the physiological measures (day and night HRV) and the seven psychosocial measures for each of the four time periods. Scores for the psychosocial measures were transformed according to the respective manual guidelines for comparative purposes (Table 2).

**Table 1:** Wilcoxon Signed Ranks Test (*z*-scores) on transformed scores for the DASS21, EUROHIS-QOL 8-item, and CBI across the four time periods (t_1_–t_4_)

Measures	*z*-*scores*		
	t_2_–t_1_	t_3_–t_2_	t_4_–t_3_
	(*n* = 15)	(*n* = 15)	(*n* = 15)
DASS21			
Stress	−.20	−.40	−.77
Anxiety	−.76	−.18	−.04
Depression	−.03	−.85	−.63
EUROHIS-QOL 8-item	−1.12	−.67	−1.66
CBI			
Person	−46	−2.33*	−.25
Work	−1.07	−1.66	−1.52
Client	−2.06*	−1.18	−.28
HRV			
Day	−1.19	−.88	−.10
Night	−.68	−1.71	−.28

Some participants did not complete all measures

* *p* < .05

**Table 2:** Means and standard deviations (SDs) for the transformed scores for DASS21, EUROHIS-QOL 8-item, and CBI across the four time periods (t_1_–t_4_)

Measures (*n* = 17, see note 1)	t_1_	t_2_	t_3_	t_4_
DASS21				
Depression				
Mean	.61	.59	.71	.73
SD	.54	.35	.55	.73
Anxiety				
Mean	.90	.70	.69	.77
SD	.76	.94	.27	.97
Stress				
Mean	1.68	1.57	1.75	1.57
SD	.94	.97	1.14	.88
EUROHIS-QOL 8-item				
Mean	3.60	3.77	3.75	3.94
SD	.66	.63	.66	.68
CBI				
Person				
Mean	46.09	45.83	39.17	38.89
SD	17.77	15.35	16.12	16.94
Work				
Mean	44.64	49.05	46.19	41.67
SD	14.11	13.60	12.55	11.97
Client				
Mean	24.48	33.89	24.67	29.29
SD	12.06	16.73	10.77	9.75
HRV (rMSSD-values)				
Day				
Mean	30.17	27.79	24.54	29.18
SD	12.18	9.34	8.94	18.65
Night				
Mean	52.69	53.42	43.48	46.61
SD	22.27	22.82	15.43	23.32

Number of complete cases at each time period was *n* = 17; it was noted that three students at different time periods did not complete the series of tests

All DASS scores are on the ‘normal’ range as presented in the guidelines for the DASS-21 [13]

EUROHIS-QOL 8-item scores within the ‘normal’ range as presented in the literature [14]

CBI scores within the ‘normal’ range (as presented in the guidelines for the CBI [15]

HRV comparison data were available although some inconsistencies are noted: nonetheless these values lie with the range of those cited by Task Force of The European Society of Cardiology and The North American Society of Pacing and Electrophysiology [12] and are consistent with other studies [9]

## Results

Seventeen junior doctors in their first year (response rate of 52 %) participated in the present research of whom 65 % were women. Most doctors (*n* = 12, 71 %) were aged between 20 and 24 years, three doctors were aged between 25 and 29 years and two doctors were aged over 30 years old.

Three pairwise comparisons were computed (Table 1) between time periods 1 and 2, time periods 2 and 3, and time periods 3 and 4. The findings indicated two (from a possible 24) statistical differences (*p* < .05) for responses to the Copenhagen Burnout Inventory for the time periods 1 and 2 (client differences, *z* = −2.06) and 2 and 3 (person differences, *z* = −2.33). No other statistical differences were noted.

When all psychosocial measures were compared with published norms (Table 1), the findings showed that this sample of junior doctors generated scores that were within the normal ranges for all self-report measures. In terms of the HRV values, the Task Force standards [12] reported a normal value range of rMSSD (27 ± 12) and the values for the day readings cited in this study lay within that range. Other data also suggested that these values are within the normal range [9]. Furthermore, there were data to support a higher rMSSD at night as found in this study [9]. Correlations between the psychosocial measures and the HRV measure (rMSSD) yielded only three significant (*p* < .05) correlations (from a possible 56). When we applied a Bonferroni correction for the multiple comparisons, these differences were non-significant.

## Discussion

This study investigated the well-being of junior doctors in their first year in a public teaching hospital in Auckland, New Zealand. The findings in this study suggest that two major inferences can be made. Firstly, the findings show that the self-reported psychosocial perceptions and the HRV measures did not fluctuate over time and the obtained measures from the junior doctors in this study were all within their respective normal ranges. Secondly, no significant correlations between the physiological data (HRV) and psychosocial measures were found over the four time periods.

The first inference did not support our hypothesis suggesting that junior doctors in this study would experience psychosocial and physiological changes over time and that these scores would show potential anomalies. Brown et al. [4] reported similar low levels of depression, anxiety and stress in their participants, although they found that critical incidents such as breaking bad news elevated heart rate and heart rate variability measures. In their study, Willcock et al. [3] reported that their sample of newly graduated medical doctors in Australia exhibited high levels of psychological distress and higher scores were found at later time periods. The difference between these findings and the current one may suggest that when obtaining the current measures the timing was not refined enough to adequately capture the stress-inducing periods that the junior doctors in this hospital may experience. It may also suggest that this group of junior doctors were in fact cognisant of their personal health and wellbeing, and thus not experiencing problems associated with accumulative stress and burnout, which may in part be explained by the proactive approach towards personal wellness driven by major stakeholders such as the Medical Council of New Zealand [5]. Evidently more research needs to be conducted with respect to the well-being of junior doctors in New Zealand over their training period.

The second inference was also unexpected and did not support our hypothesis that psychosocial perceptions would be significantly associated with HRV measures. The non-significant correlations between the physiological data (HRV) and the psychosocial measures indicate that changes in HRV are not consistent with changes in perception of well-being. This finding may support the claim of mind–body dissonance suggesting that doctors engage in avoidant behaviour or utilise denial coping strategies or may not wish to be seen as vulnerable [2]. This claim is reasonable given that medicine is often perceived and documented as a stressful occupation [1, 2, 5, 7]. In their study, Gander et al. [7] found that 13 % of those junior doctors surveyed were working over 70 h per week and only 30 % were working less than 50 h per week. In addition, they found that “participants were twice as likely as the general adult population to score as excessively sleepy” [7]. Furthermore, these participants were more prone to fatigue-related clinical error compared with their more senior medical colleagues. In their review, Wallace et al. [2] presented a strong argument for “suboptimum attention to self-wellness by physicians” which is likely to be greater among junior colleagues. In their model, Wallace et al. showed clear links between the experience of stressful outcomes as a consequence of experiencing workplace stressors and indifference to personal self-care. Further research in this area is seen as being crucial given the discrepancies in the literature, the current findings and those expected.

We further acknowledge certain limitations associated with this study. First, the hospital environment does not simulate an ideal research world and hence asking junior doctors to wear HRV equipment was seen as inconvenient or time consuming and this likely hampered our collection of data due to either participant drop-out or non-participation. Next, even though all medical personnel working with these doctors were not able to access the data, the notion of informed consent implies that the participants knew the aims of the study and those involved, and this may have biased their responses. In addition, the low sample size hindered our statistical methods and adversely affected the power of the statistics and this hampered our ability to consider sub-groupings and potential confounders such as the clinical runs these doctors attended. Lastly, the study participants were recruited from only one hospital which could limit generalizability of the results.

## Conclusion

The evidence in this study suggest that this group of first year junior doctors were not experiencing problems associated with depression, anxiety, stress, burnout and/or HRV. What is of further of interest is that the responses from the self-report instruments measuring stress, anxiety, depression, quality of life and burnout did not consistently correlate with the HRV information suggesting a mind–body disconnection. To further explore the incongruity between the current findings and those found elsewhere, we suggest using a larger sample and establishing more rigorous and reasonable research protocols that would work with doctors’ workplace constraints.
